# Red Tide Phenomenon Leading to Panic Attack and Mass Casualty among Children in Coastal Kerala

**DOI:** 10.4103/0970-0218.66880

**Published:** 2010-04

**Authors:** KE Elizabeth, H Gopakumar, Gibby Koshy

**Affiliations:** Department of Pediatrics, SAT Hospital, Medical College, Thiruvananthapuram, Kerala, India

## Introduction

Red tide phenomenon is an event that occurs with varying intensity almost every year.(1) This occurs along the coastal areas. This is frequently described along the Florida coast and Gulf of Mexico.(2) The timing of the phenomenon is difficult to predict. This occurs almost every month along the coast of Florida. The waste products and decaying matter along the seashore get washed into the sea especially during rains. These nutrients washed from the coastal areas into the sea water are consumed by the unicellular phytoplankton algae, Cochlodeneum Krikoids, which forms the lower most organism in the food chain.(3) The right salinity, temperature and nutrients are thought to nourish these organisms. These may proliferate seasonally and reach enormous amounts thereby discoloring the water surface; hence this phenomenon is also referred to as ’Dinoflagellate boom’. Most species produce red pigments and so this phenomenon is often referred to as red tide phenomenon. Some produce reddish brown, yellow or green pigments as well.(3) The phenomenon though referred to as red tide phenomenon, it has nothing to do with tidal activity. These algae are usually harmless, but some of them may produce very potent toxins. With massive proliferation, they may deplete the seawater of oxygen leading to death of fishes in water. Usually fishes that dwell in deep waters are affected. Following widespread death of fishes, there may be release of noxious gases. Inhalation of these gases by human beings can cause clinical manifestations such as syncopial attacks, headache, drowsiness, abdominal pain, nausea, vomiting and panic attacks.(4) However, it is interesting to note that the panicky situation is rarely associated with significant morbidity or mortality.(5) The algae are also consumed by shell fishes which when consumed by human beings can cause gastrointestinal manifestations. Hence human beings are advised to abstain from consuming certain harmful shellfishes during the phenomenon. During red tides, the dinoflagellate, Ptychodiscus Brevis is known to elaborate several neurotoxins such as saxitoxin that inhibit the sodium potassium pump and nerve conduction. Filter feeding molluscs concentrate these toxins and ingestion of these shellfishes may lead to poisoning. Previously, there was a similar event in the same locality with significant morbidity and a few mortality (unpublished).

## Case Series

During the recent red tide phenomenon that occurred along the coastal strip of Southern Kerala in mid September 2004, 67 children were admitted. The admitted children were attending school assembly at St. Roth School, Valiyathura when they inhaled a pungent stinging gas. The age and sex distribution is given in Table 1.

**Table 1:** Age and sex distribution

Age Group	Male		Female		Total	%
	Number	Percentage	Number	Percentage		
<10 years	2	2.98	15	22.3	17	25.28
10-15 years	4	5.97	46	68.65	48	74.62

Total	6	8.95	61	91.44	67	100

The common symptoms were nauseating smell (100%), giddiness (13%), vomiting (10%) and abdominal pain (6%) [Table 2]. 7% of the children required ICU admission and observation. Intravenous fluids was required in 6% of cases.

**Table 2:** Distribution according to clinical manifestations

	No	Percentage
Nauseating smell	67	100
Nausea	9	13
Vomiting	4	6
Abdominal pain	4	6
Fever	2	3

## Discussion

75% of children were above 10 years in the present case series. It is interesting to note that majority of the children were females ( 91 % ). This may point to an increased vulnerability of females to the ’mass effect”. Very young children were not admitted probably because they were staying indoors during the phenomenon. The most common clinical manifestation was nauseating smell, which was present in 100% of the cases, followed by giddiness, nausea and vomiting. 6% of the cases required intravenous fluids. The majority of cases required only observation and reassurance. The mean hospital stay was two days. Investigations like complete blood count, urine analysis, renal function test, serum electrolytes and liver function test done in selected cases were normal. There were no mortality or significant morbidity associated with the phenomenon. The children required only symptomatic management. Those with gastrointestinal symptoms and syncope were treated with intravenous fluids and Ranitidine and those with fever were given paracetamol. Majority of them required no treatment except for observation and monitoring of vital signs.

## Conclusion

This communication is an attempt to sensitize the medical professionals as well as the public regarding the generally harmless, self-limiting nature of the red tide phenomenon.
